# Recognition and management of abdominal compartment syndrome among German pediatric intensivists: results of a national survey

**DOI:** 10.1186/2110-5820-2-S1-S8

**Published:** 2012-07-05

**Authors:** Torsten Kaussen, Gerd Steinau, Pramod Kadaba Srinivasan, Jens Otto, Michael Sasse, Franz Staudt, Alexander Schachtrupp

**Affiliations:** 1 Department of Neonatology and Pediatric Intensive Care, Children's Hospital Dritter Orden, Bischof-Altmann-Str. 9, 94032 Passau, Germany; 2 Department of Pediatric Cardiology and Intensive Care Medicine, University Children's Hospital, Medical University Hannover (MHH), OE 6730, Carl-Neuberg-Str. 1, 30625 Hannover, Germany; 3 Department of Surgery, University Hospital RWTH Aachen, Pauwelsstr. 30, 52074 Aachen, Germany; 4 Institute for Laboratory Animal Science and Experimental Surgery, University Hospital RWTH Aachen, Pauwelsstr. 30, 52070 Aachen, Germany

**Keywords:** intra-abdominal pressure, intra-abdominal hypertension, abdominal compartment syndrome, children, intensive care unit, questionnaire, decompressive laparotomy.

## Abstract

**Introduction:**

Several decades ago, the beneficial effects of goal-directed therapy, which include decompressive laparotomy (DL) and open abdomen procedures in cases of intra-abdominal hypertension (IAH) in children, were proven in the context of closures of abdominal wall defects and large-for-size organ transplantations. Different neonatologic and pediatric disease patterns are also known to be capable of increasing intra-abdominal pressure (IAP). Nevertheless, a considerable knowledge transfer regarding such risk factors has hardly taken place. When left undetected and untreated, IAH threatens to evolve into abdominal compartment syndrome (ACS), which is accompanied by a mortality rate of up to 60% in children. Therefore, the present study looks at the recognition and knowledge of IAH/ACS among German pediatric intensivists.

**Methods:**

In June 2010, a questionnaire was mailed to the heads of pediatric intensive care units of 205 German pediatric hospitals.

**Results:**

The response rate was 62%. At least one case of IAH was reported by 36% of respondents; at least one case of ACS, by 25%. Compared with adolescents, younger critically ill children appeared to develop IAH/ACS more often. Routine measurements of IAP were said to be performed by 20% of respondents. Bladder pressure was used most frequently (96%) to assess IAP. Some respondents (17%) only measured IAP in cases of organ dysfunction and failure. In 2009, the year preceding this study, 21% of respondents claimed to have performed a DL. Surgical decompression was indicated if signs of organ dysfunction were present. This was also done in cases of at least grade III IAH (IAP > 15 mmHg) without organ impairment.

**Conclusions:**

Although awareness among pediatricians appears to have been increasing over the last decade, definitions and guidelines regarding the diagnosis and management of IAH/ACS are not applied uniformly. This variability could express an ever present lack of awareness and solid prospective data.

## Introduction

The problem of intra-abdominal hypertension (IAH) that resulted from an operative closing of a birth defect in the abdominal wall has been recognized for decades in pediatric surgery [1]. Therefore, gastroschisis and omphalocele are regarded as prototypes of illnesses causing IAH [2,3]. As early as the mid-twentieth century, various expanding abdominoplasties and staged abdominal wall closure techniques [4-7] enabled the development of therapy options to help prevent the deleterious consequences of an increase in intra-abdominal pressure (IAP) and its transition into abdominal compartment syndrome (ACS). This development has expanded into pediatric transplant surgery and has decreased morbidity as well as mortality [8-10]. In the 1970s and 1980s, indirect procedures for measuring IAP via the stomach, rectum, and bladder were established [11-13]. These procedures were developed to ensure intra-operative objectivity. By applying them, a pressure-dependent decision on further therapy has become possible, leading to a further improvement in prognosis. From animal testing and studies performed on adults, it has become clear that a series of other surgical and internistic diseases as well as certain risk factors can also lead to the development of IAH [14-16]. In spite of this progress, pediatric medicine has yet to experience an essential knowledge transfer regarding other illnesses that potentially increase patient risk. This is unfortunate insofar as standardized diagnostics and therapy in adults have recently been shown to reduce the mortality rate by 50% [17]. In line with this, it would be desirable to unmask all risk factors and illnesses also in children. Furthermore, increasing the acceptance of a strict realization of comparable standard operating procedures should be a goal.

The main purpose of this national survey was to achieve an overview of the current situation in Germany. In addition, it aimed to describe the level of awareness concerning IAH and ACS. Moreover, the survey endeavored to evaluate potential risk factors as well as current diagnostic and management modalities in practice.

## Methods

In June 2010, a questionnaire (see Additional file 1) was mailed to the heads of all pediatric intensive care units (PICUs) of 205 German pediatric departments with an established pediatric residency program. The chair of the department or the senior physician heading the intensive care unit (ICU) was contacted personally via letter. The performance of the present survey was self-financed by the authors. Financial aid was neither offered nor accepted. The investigational period lasted for 10 weeks. A reminder was sent to the units that have failed to respond within 6 weeks. Overall, 360 surveys were distributed. Each double-sided survey contained 16 groups of questions (see Additional file 1) which had to be answered by entering text or multiple choice. Incomplete questionnaires were included.

Results were entered into an Office Excel 2003 SP3 database (Microsoft^® ^Germany GmbH, Unterschleißheim, Germany) and analyzed descriptively. Results are given as absolute numbers and percentages, as well as mean with standard deviation when appropriate. To meet the anatomical and (patho)physiological requirements of different age groups and their concomitant weights and sizes, children are divided into five age groups: 'preterm and newborn' (aged up to 28 days), 'baby/infant/suckler' (29 days to < 1 year), 'toddler' (> 1 to < 6 years), 'pupil' (> 6 to < 12 years), and 'adolescent' (> 12 to < 18 years). The theoretical basis for answers to questions concerning definitions as well as therapeutical options concerning IAH/ACS in children is provided by the World Society of the Abdominal Compartment Syndrome (WSACS) consensus definitions and recommendations, which were adapted to make them partly child oriented by Malbrain et al. and Ejike et al. (Tables 1 and 2) [18,19].

**Table 1:** Child-oriented adapted WSACS consensus definitions

Condition	Definition
IAP	Pressure within the abdominal cavity (in millimeters mercury; measured at end expiration)
Normal IAP	7 ± 3 mmHg in critically ill children
APP	APP = MAP - IAP
IAH	Sustained or repeated pathological elevation in IAP &#8805 10 mmHg
Grade I	IAP 10 to 12 mmHg
Grade II	IAP 13 to 15 mmHg
Grade III	IAP 16 to 18 mmHg
Grade IV	IAP greater than 18 mmHg
ACS	Sustained IAP ≥ 10 mmHg associated with new organ dysfunction/failure
Primary ACS	Condition associated with injury or disease in the abdominopelvic region
Secondary ACS	Condition that does not originate from the abdominopelvic region
Recurrent ACS	Condition in which ACS redevelops after previous surgical or medical treatment of primary or secondary ACS

APP, abdominal perfusion pressure; MAP, mean arterial pressure; IAP, intra-abdominal pressure. Modified after Ejike et al. [19].

**Table 2:** Therapeutical options to lower IAH

Options	Evacuation of intraluminal contents	Evacuation of intra-abdominal space occupying lesions	Improvement of abdominal wall compliance	Optimization of fluid administration	Optimization of abdominal (APP) and systemic perfusion
Medical, non-invasive options	Gastric/rectal tube Diet		Analgetics and sedatives	Modest fluid administration	Goal-directed fluid administration
	Prokinetics		Positioning	Diuretics	Pressors
	Fasting		Muscle relaxants		Inotropes
Interventional, minimal-invasive options	Gastric decompression	Paracentesis		Continuous venous hemofiltration	
	Colonoscopic decompression	Percutaneous catheter drainage			
Surgical, invasive options		Decompressive laparotomy	Escharatomy/fasciotomy		Laparostomy (TAC)

TAC, temporary abdominal closure. Adapted from Ejike et al. and Cheatham et al. [19,50]

## Results

Of the 360 questionnaires distributed, 127 replies were received (41.6%). Larger pediatric departments with more than one specialized ICU returned only one questionnaire representing the current status of all wards. As a result, a response rate of 62.0% was achieved (127/205). Participants were asked to describe the structure and orientation of their ICU as well as the level of medical care (Table 3).

**Table 3:** Descriptive statistic concerning the structure of answering clinics

Factor	Structure and orientation of ICU	Percentage (%)
Administrative affiliation of the ICU (*n *= 127)	P PS A P + PS collective A + PS collective	91 6 1 1 1
Medical focus of the ICU (*n *= 115)	NICU rather than PICU PICU rather than NICU Exclusive NICU Exclusive PICU No answer	63 11 19 1 6
Age distribution of treated patients (*n *= 112)	Neonatologic Pediatric	70 30
Level of medical care at participating NICUs (*n *= 113)	High level Intermediate level Low level No answer	61 18 15 6
Size of ICU/Number of cases in 2009 (*n *= 112)	< 351 patients/year 351 to 700 patients/year > 700 patients/year	30 41 29
Part of university hospitals (*n *= 118)		27

A, anaesthesiology; NICU, neonatal intensive care unit; P, pediatrics; PICU, pediatric intensive care unit; PS, pediatric surgery.

### Awareness and diagnosis of IAH and ACS

Almost half of all ICUs that responded (56/123) stated that IAH and ACS are present in everyday clinical practice (B.1 of Table 4). At least one case of IAH was noted by 36% (44/124) in 2009 and at least one case of ACS, by 26% (32/124) (B.3 of Table 4). Preterms and newborns seem to be affected in nearly 20% of cases (12/61), and about 13.5% are at risk of developing ACS (8/60). In contrast, 'only' 3 to 5 out of 100 toddlers, pupils, and adolescent patients were claimed to be affected (B.4 of Table 4).

**Table 4:** Distribution of responses

Question	Stated question and choices	Answers (%)
B.1	Occurrence and relevance of IAH/ACS in clinical practice	
	• Never	54 (67/123)
	• Seldom	39 (48/123)
	• Regularly	6 (7/123)
	• Often	1 (1/123)
	Decade of first-time diagnosing IAH/ACS:	
	• Before 1980	2 (1/45)
	• 1980 to 1989	4 (2/45)
	• 1990 to 1999	40 (18/45)
	• 2000 to 2009	53 (24/45)
B.2	Awareness of current WSACS-definitions (tested by free text)	
	• principle of IAH definition correctly described (increased IAP)	43 (21/49)
	• principle of ACS definition correctly described (IAH + organ dysfunction)	35 (17/49)
	Stated IAP thresholds for IAH	
	• IAP ≥ 10 mmHg	42 (5/12)
	• IAP ≥ 12 mmHg	25 (3/12)
	• IAP ≥ 15 mmHg	25 (3/12)
	• IAP ≥ 20 mmHg	8 (1/12)
B.3	Frequency of diagnosed IAH at answering ICUs in 2009	
	• 0 times IAH	64 (79/124)
	• 1 to 10 times IAH	30 (37/124)
	• > 10 times IAH	6 (7/124)
	Frequency of diagnosed ACS at answering ICUs in 2009	
	• 0 times ACS	75 (93/124)
	• 1 to 5 times ACS	24 (30/124)
	• > 5 times ACS	1 (1/124)
	Distribution of causes of ACS	
	• Primary ACS	45 (16/35)
	• Secondary ACS	49 (17/35)
	• Not distinguishable	6 (2/35)
B.5	Awareness and use of current WSACS definitions (tested by multiple choice)	
	• IAH definition correctly chosen (increased IAP)	4 (5/124)
	• ACS definition correctly chosen (increased IAP + new organ dysfunction)	17 (22/124)
	Clinical symptoms stated to be associated with increased IAP in children	
	• Oliguria to anuria	20 (33/169)
	• From peritonism, to peritonitis, and to acute abdomen	15 (26/169)
	• Abdominal distension	14 (24/169)
	• Hemodynamic insufficiency	14 (24/169)
	• Respiratory insufficiency	12 (20/169)
	• Organ dysfunction (including ileus)	11 (19/169)
	• Radiologic findings	8 (13/169)
	• Impaired venous reflux to increased central venous pressure	5 (8/169)
	• Others	1 (1/169)
B.6	Share of respondents stating to measure IAP regularly	20 (25/125)
	Stated reasons for not measuring IAP	
	• Clinical diagnosis (IAP measurement not necessary)	48 (48/100)
	• Lack of technical equipment	42 (42/100)
	• Lack of therapeutical consequence	11 (11/100)
	• Fear for invasiveness	9 (9/100)
	• Fear for infection	5 (5/100)
	• Fear for additional expenditure	5 (5/100)
	Frequency of measurements among those who stated to measure IAP	
	• once per day	31 (7/23)
	• two times per day	17 (4/23)
	• three to four times per day	17 (4/23)
	• Continuously (or more than four times per day)	35 (8/23)
	• In cases of clinical signs of IAH or ACS	70 (16/23)
	• In cases of organ dysfunction or failure	17 (4/23)
B.7	Predominantly used *indirect *IAP measurement methods	
	• via intra-vesical pressure	96 (24/25)
	• via intra-gastric pressure	24 (6/25)
	• via PIP (PIP increase is a consequence of IAH)	16 (4/25)
	• via central venous pressure	4 (1/25)
	Predominantly used *direct *IAP measurement methods	
	• via Spiegelberg^® ^probe^a ^(modified brain pressure probe)	4 (1/25)
	• via CAPD catheter	4 (1/25)
	• via surgical drainage	4 (1/25)
	• via intra-abdominal placed cardiac catheter	4 (1/25)
B.8	Share of respondents who stated they would measure IAP more often if the procedure and technical requirements became easier and more standardized	68 (60/88)
B.12	Share of respondents having performed at least one decompressive laparotomy in 2009	20 (26/127)
	Stated survival rate of ACS patients in 2009	
	• Surgically treated children	88 (18/20)
	• Non-surgically treated children	71 (5/7)
	Share of respondents who would surgically decompress again (if indicated)	100 (26/26)

CAPD, continuous abdominal peritoneal dialysis; PIP, peak inspiratory pressure. ^a^Spiegelberg KG, Hamburg, Germany.

### Risk factors for IAH and ACS

**Figure 1:**
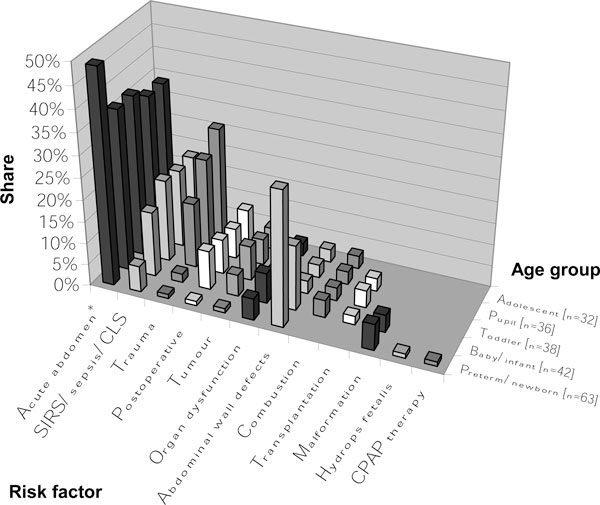
**Risk factors for IAH/ACS among children dependent on the age group**. Respondents were asked to mention disease patterns which, to their experiences, most often cause IAH/ACS in children of different age classes (*n *= 32 to 63; percentage of given answers). 'Abdominal wall defects' consist of gastroschisis, omphalocele, and diaphragmatic hernia. 'Organ dysfunction' subsumes cardiac insufficiency as well as hepatic and renal dysfunction or failure. 'Postoperative' includes abdominal, cardiac, and thoracic surgery. CLS, capillary leak syndrome; CPAP, continuous positive airway pressure; SIRS, systemic inflammatory response syndrome. ^a^The different disease patterns which are summarized with 'acute abdomen' are more detailed in Figure 2.

**Figure 2:**
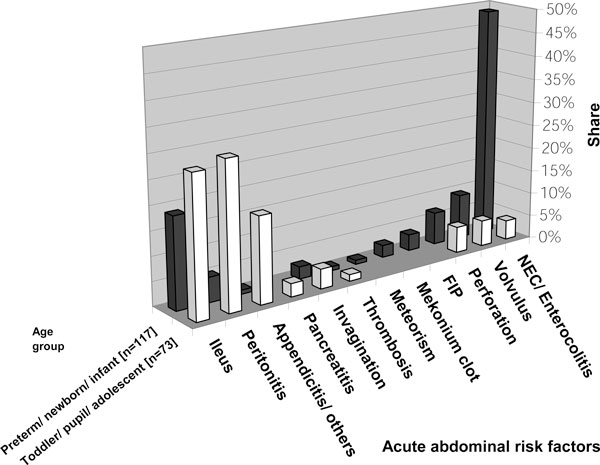
**Risk factors for IAH-/ACS-inducing acute abdomen among children**. Respondents were asked to mention disease patterns which, to their experiences, most often cause IAH/ACS in children (Figure 1). Dependent on the age class, different 'acute abdominal risk factors' were mentioned (*n *= 32 to 63; percentage of given answers). Dependent on the age causes might be divided into two clusters (neonatal vs. pediatric). FIP, focal intestinal perforation; NEC, necrotizing enterocolitis.

Asked to state disease patterns which frequently induce IAH, intensivists most often indicated illnesses accompanying the clinical picture of an 'acute abdomen' (Figure 1). The presumed underlying causes of acute abdomen, however, varied essentially between younger and older children (Figure 2). Trauma was reported as the underlying main reason for older pediatric patients. Regardless of age group, other reported causes of IAH were systemic inflammatory response syndromes without or with bacteraemia (sepsis) as well as capillary leak syndromes (Figures 1 and 2).

### Definition and diagnosis of IAH and ACS

No more than 3.9% (5/127) of respondents chose IAH/ACS diagnosis criteria, and no more than 16.5% (21/127) chose definitions (B.5 of Table 4) in accordance with the current literature (Table 1). The majority of respondents (99/124) stated that clinical signs exclusively provide the basis for their diagnosis of 'IAH' and 'ACS'. Asked to name symptoms regularly associated with IAH, intensivists most often cited changes of abdominal tension and perimeter (29.6%) as well as impairment of diuresis (19.5%) and hemodynamics (14.2%) (B.5 of Table 4).

### Measurement of IAP

When asked whether IAP was measured, 20% of respondents (25/125) answered with yes. Reasons for not measuring IAP are depicted in Table 4. Of the 25 respondents claiming to measure IAP, only 23 delivered further information about their measurement standards (B.6 of Table 4). The cutoff for IAH in children was considered 10 mmHg by 42% of respondents (5/12; B.2 of Table 4). Bladder pressure measurements (24/25) were performed most often to quantify IAP ('gold standard') [11]. Other methods only played a minor role (B.4 of Table 4). The majority, in this case 68.2% of respondents (60/88), stated that they would measure IAP more often if the procedure and technical requirements became easier and more standardized (B.8 of Table 4).

### Therapeutical strategies concerning the management of IAH/ACS patients

**Figure 3:**
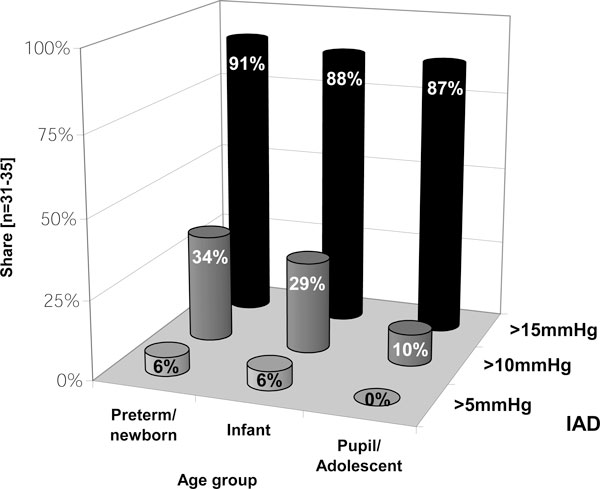
**Critical IAP threshold used for surgical decompression dependent on the age of patient**. Respondents were asked to mention at which IAP level surgical decompression would be taken into consideration if children of different age classes would be affected (*n *= 31 to 35; percentage of given answers).

**Figure 4:**
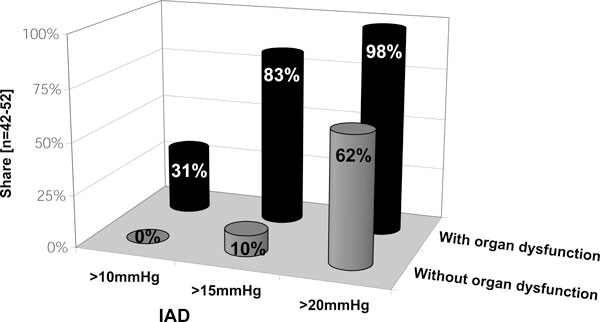
**Critical IAP threshold used for surgical decompression dependent on organ (dys)function**. Respondents were asked to mention at which IAP level surgical decompression would be taken into consideration depending on the absence or presence of organ dysfunction (*n *= 42 to 52; percentage of given answers).

Unlike that of older children, the likelihood of young pediatric patients undergoing more invasive therapy options earlier appears to be inversely related to their age (Figure 3). As long as organ dysfunction remains absent, more invasive therapy options (Table 2) are stated as not being used until IAP exceeds 20 mmHg. In contrast, as soon as organ function deteriorates, respondents appear to decompress much earlier, even at moderate IAP elevations (Figure 4). Experiences and opinions of respondents concerning decompressive laparotomy (DL) are summarized in Table 4 (B.12).

## Discussion

In contrast with other pediatric diseases with high risk, the problem of IAH in newborns with congenital abdominal wall defects and in children with a need for large-for-size organ transplants appears to become more and more well known. Nevertheless, the results of the present survey seem to emphasize the impression that there is still a considerable lack of knowledge, awareness, and goal-oriented therapy.

### Literature overview

So far, only two study groups conducted surveys that focused on pediatric health care providers. During two pediatric congresses (2006 and 2007), Ejike et al. handed out 1,107 questionnaires and received 517 replies (return rate 46.7%) [20]. In contrast to our survey, not only pediatric intensivists (30.2%), but also general pediatricians (3.9%) and pediatric nurses (59.8%) participated. Within the context of another congress in 2010, the same study group repeated this survey among pediatric nurses and compared the results of both surveys with each other (return rate 62.7% (433/691); [21]).

In 2001, Kimball et al. sent a survey to 4,538 members of the Society of Critical Care Medicine with a response rate of 35.7% [22]. Of the 1,622 respondents, 57 physicians stated that they worked in a PICU (3.5%), and 294 had primary training in pediatrics (18.1%).

Thus, to the best of our knowledge, the present study is the first to reflect the results of a nationwide survey conducted among pediatric intensivists without bias that is a result of a dependency on different professional groups and congress participants or membership in certain societies.

### Awareness and incidence of IAH and ACS

Pediatricians seem to have the impression that IAH occurs more in younger patients. In accordance to our results, Akhobadze et al. found IAH in 18.1% of neonatal cases [23]. For the rest, the incidence of ACS in children is estimated to be 0.6% to 9.8% [19,24-26]. The best possible explanation for the varying risk could be the different anatomical and physiological circumstances.

### Risk factors for IAH and ACS

Each intensivist should also know rare risk factors in order to be able to adequately monitor and medicate at-risk patients (Figures 1 and 2). Risk factors vary substantially depending on the age of pediatric patients; therefore, they may be divided into *congenital *and *acquired *causes as well as into *neonatal *and *pediatric *origins. While among neonates, abdominal wall defects and necrotizing enterocolitis appear to play a major role; the at-risk profile moves in the direction of trauma among older children.

The survey of Kimball et al. [22] revealed similar underlying disease patterns. They found 'third spacing' and large volume resuscitation, as well as trauma accompanied by bleeding, to be the most important risk factors. Accordingly, Beck et al. argued that children develop IAH more often on the basis of ischemia-reperfusion states [24]. Consistent with the results of a study performed by Ejike et al. [25], our results exhibit a trend towards secondary ACS (Tables 1 and 4 (B.3); Figure 1). Further risk factors are described in Figures 1 and 2; however, they are not complete.

### Definition and diagnosis of IAH and ACS

Contrary to current definitions, half of the respondents stated that IAH and ACS would be diagnosed using clinical criteria alone. This was despite the fact that neither clinical exam nor abdominal perimeter has proven to be accurate parameters and delivers a sensitivity of no more than 50% [27-30]. In the study by Kimball et al., 20% of respondents relied solely on the clinical picture [22], and only 7.2% based the diagnosis of IAH on IAP measurement. This result is similar to the one found in our study. In the investigation of Newcombe et al., the percentage of understanding the correct definition of ACS even dropped when compared with the pre-study of Ejike et al. [21]. At that time, the definition of ACS had been mistaken for that of IAH by 53.2% of their participants [20]. All of these results imply that there is still 'confusion among pediatric health care providers' [20]. They further imply that patients are not being diagnosed correctly and, therefore, are receiving inadequate treatment. Deeming critically ill patients at risk of developing IAH without measuring IAP seems highly dangerous.

### Measurement of IAP

In fact, only 20% of our respondents regularly measure IAP compared with 24.2% in the investigation of Ejike et al. [20]. According to Steinau et al. [31], who recommended four to six hourly control measurements in cases of impending IAH in children, IAP is measured at least six hourly by about 50% of participants claiming to perform measurements. Among them, 17% stated that they measure it continuously. Unfortunately, respondents did not note which kind of technique they used. Commercially available continuous pressure measurement systems [32,33] appear to display rather an exception than the rule in children.

Regardless of whether measurements are performed continuously or intermittently, indirect methods via the bladder (gold standard) [11] and the stomach are most often used in children. This is in accordance with former ACS surveys [20-22,34-40]. All measuring techniques mentioned (B.7 of Table 4) have been examined for their ability to reflect the IAP in children [12,13,41-44].

### Therapeutical strategies concerning the management of IAH/ACS patients

In cases of imminent or existent IAH, several medical, interventional, and surgical therapy options are available (Table 2). Nonetheless, in the absence of appropriate standards, there seems to be great insecurity regarding which kind of therapy is necessary in which diagnostic constellations.

In cases of persisting or even progressing IAH, all available therapy options should be applied courageously, in an escalating way, without wasting any time. Surprisingly, minimal-invasive decompressive methods, such as peritoneal drainage and paracentesis, seemed to play a minor role and were named in no more than 15% of answers. This was the case although several authors proclaimed their beneficial merits in lowering IAH and avoiding the need for surgical decompression [41,45-48].

The readiness to decompress surgically is strongly dependent on the age of the patient and the presence of organ dysfunction. When younger children are affected and in cases of ongoing organ failure, intensivists appear to be willing to use invasive therapy forms earlier. This is in accordance with the findings made by Kimball et al. [22]. When one considers their results, pediatric intensivists seem to have become more familiar with invasive rescue therapies over the last decade. This might be a sign of a deeper comprehension of the detrimental pathophysiological effects of IAH. In comparison with adults, however, invasive procedures are the absolute exception and applied much less. Whether this can be traced back to a special reluctance among pediatricians and pediatric surgeons cannot be determined using this survey. Further, it cannot be determined whether the majority of underlying pediatric illnesses can be treated successfully in an either non-invasive or minimally invasive way. Nevertheless, it provides arguments for the idea that an aggressive therapy, insofar indicated, can improve the prognosis.

### Prognosis of children suffering from ACS

Mortality from ACS accounts for 22% to 60% in children [23-26,31]. When left untreated, brisk, progressing multi-organ failure (MOF) is said to be an essential underlying reason. Ejike et al. [25] reported a mortality rate of 50% among critically ill children suffering from primary ACS, 80% of whom had undergone a DL. In contrast, mortality in a secondary ACS group was 67%, 11% of which had undergone decompression. They concluded that mortality among primary ACS patients might be lower because laparotomy is performed earlier, not for the sake of IAH but rather for the sake of the underlying intra-abdominal disorder. Indirectly, this might imply that timely decompression is able to lower morbidity and mortality. Accordingly, the stated survival rate in the present study was lower among surgically treated children when compared with non-invasively treated patients. However, there might be a limited timeframe in which decompression really is able to improve the outcome. If an inflammatory 'point-of-no-return' is surpassed and MOF takes on a life of its own, even decompression is no longer able to prevent death anymore, as shown by De Waele et al. [49].

### Forward-looking statements

The goal of future research must be to develop a standardized diagnostic as well as a therapy algorithm also for children. An initial algorithm was introduced by Steinau et al. [31]; however, it does not yet contain the IAP limits adapted for children that were suggested by Ejike et al. [19]. That these limits reflect the experience of pediatricians practicing in German hospitals is also shown in the results of our survey. Almost half of all respondents saw an IAP of 10 mmHg as a critical pressure limit for children, which is clearly below the limits for adults defined by the WSACS [18]. That pediatric IAP thresholds must be substantially lower than those used in adults is pathophysiologically explainable by the fact that blood pressures in children and especially in neonates are vastly lower than those in adults. Therefore, also a mild increase in IAP is able to substantially impair local as well as systemic circulation and organ perfusion. Based on the definitions and recommendations published by Ejike et al. [19], the authors therefore suggest a new scale division concerning different grades of ACS in children (Table 1).

Since it has been shown that indicators as well as risk factors, illnesses, and critical abdominal pressure appear to vary considerably depending on pediatric patient age, an algorithm adapted for each age class must be developed. Further, since our results have shown that the level of skepticism concerning methods for measuring IAP continues to be high in spite of the positive experiences in neonatal and transplant surgery, their effectiveness and harmlessness must be supported by evidence. Only then can it be hoped that the acceptance of diagnostic and therapeutical standards increases and that they are implemented in the treatment of critically ill children.

The goal of all these efforts must be to reduce morbidity and mortality from IAH and ACS significantly by applying the appropriate algorithms. Since the introduction of such standards in the treatment of adults, the indication of ACS could apparently be drastically reduced through an early and courageous therapy for pending or advanced IAH [17].

### Limitations of this survey

Surveys are known to have limitations and represent personal experience rather than objective data. The goal of our survey was to be purely descriptive. Therefore, no absolute conclusion may be drawn using this manuscript alone. That answers were given by interviewees familiar with this particular topic and interested in the resolution of certain unsolved problems might result in a bias towards putative over-recognition.

Some questionnaires were only partially completed, decreasing the strength of the whole investigation. Nonetheless, the omission of certain items might be interpreted as an honest expression of a lack of knowledge. Besides, findings using even few participants who are more familiar with IAH/ACS should be presented in order to offer clinical examples till more evidence-based data are available.

However, data from this survey display a significant lack of consensus and certainty among pediatric intensivists. This observation might help guide future studies with a multicenter prospective randomized approach.

## Conclusion

This study describes the results of the first nationwide survey concerning the knowledge of pediatricians on IAH and ACS. Although awareness among pediatricians appears to have been increasing over the last decade, definitions and guidelines regarding diagnosis and therapy of IAH/ACS are not applied uniformly. This variability could be the expression of a continued lack of both awareness and solid prospective data. The latter might lead to the development of accepted standard operating procedures and, ultimately, to lower morbidity and mortality of children with IAH/ACS.

## Abbreviations

ACS: abdominal compartment syndrome; APP: abdominal perfusion pressure; DL: decompressive laparotomy; IAH: intra-abdominal hypertension; IAP: intra-abdominal pressure; ICU: intensive care unit; MOF: multi-organ failure; PICU: pediatric intensive care unit; WSACS: World Society of the Abdominal Compartment Syndrome.

## Competing interests

In addition to his assistant professorship at the Technical University of Aachen (Germany), Alexander Schachtrupp is head of the Department of Medical Sciences at B. Braun Melsungen in Germany. B. Braun does not distribute any medical devices or products concerning the diagnosis and/or treatment of IAH or ACS. The other authors declare that they have no competing interests.

## Authors' contributions

TK and AS mainly did literature research, data collection, and article writing. PKS delivered speaker linguistic advice. GS, FS, JO and MS reviewed the article. All authors read and approved the final manuscript.

## Supplementary Material

Additional file 1 **Postal questionnaire**. Click here for file

## References

[B1] Kidd JN Jr Jackson RJ Smith SD Wagner CW Evolution of staged versus primary closure of gastroschisis Ann Surg 2003 237 759 764 12796571 10.1097/01.SLA.0000071568.95915.DC PMC1514688

[B2] Watson RA Howdieshell TR Abdominal compartment syndrome South Med J 1998 91 326 332 10.1097/00007611-199804000-00002 9563421

[B3] DeCou JM Abrams RS Miller RS Gauderer MW Abdominal compartment syndrome in children: experience with three cases J Pediatr Surg 2000 35 840 842 10.1053/jpsu.2000.6857 10873022

[B4] Gross RE A new method for surgical treatment of large omphaloceles Surgery 1948 24 277 292 18872858

[B5] Allen RG Wrenn EL Jr Silon as a sac in the treatment of omphalocele and gastroschisis J Pediatr Surg 1969 4 3 8 10.1016/0022-3468(69)90177-8 4238305

[B6] Clark RH Walker MW Gauderer MW Factors associated with mortality in neonates with gastroschisis Eur J Pediatr Surg 2011 21 21 24 10.1055/s-0030-1262791 21328190

[B7] Schuster SR A new method for the staged repair of large omphaloceles Surg Gynecol Obstet 1967 125 837 850 4227443

[B8] Pentlow A Smart NJ Richards SK Inward CD Morgan JD The use of porcine dermal collagen implants in assisting abdominal wall closure of pediatric renal transplant recipients with donor size discrepancy Pediatr Transplant 2008 12 20 23 10.1111/j.1399-3046.2007.00824.x 18086240

[B9] Ong TH Strong R Zahari Z Yamanaka J Lynch S Balderson G Pillay P The management of difficult abdominal closure after pediatric liver transplantation J Pediatr Surg 1996 31 295 296 10.1016/S0022-3468(96)90019-6 8938363

[B10] Karpelowsky JS Thomas G Shun A Definitive abdominal wall closure using a porcine intestinal submucosa biodegradable membrane in pediatric transplantation Pediatr Transplant 2009 13 285 289 10.1111/j.1399-3046.2008.01086.x 19032420

[B11] Lacey SR Carris LA Beyer AJ III Azizkhan RG Bladder pressure monitoring significantly enhances care of infants with abdominal wall defects: a prospective clinical study J Pediatr Surg 1993 28 1370 1374 10.1016/S0022-3468(05)80329-X 8263703

[B12] Lacey SR Bruce J Brooks SP Griswald J Ferguson W Allen JE Jewett TC Jr Karp MP Cooney DR The relative merits of various methods of indirect measurement of intraabdominal pressure as a guide to closure of abdominal wall defects J Pediatr Surg 1987 22 1207 1211 10.1016/S0022-3468(87)80739-X 2964519

[B13] Banieghbal B Gouws M Davies MR Respiratory pressure monitoring as an indirect method of intra-abdominal pressure measurement in gastroschisis closure Eur J Pediatr Surg 2006 16 79 83 10.1055/s-2006-924051 16685611

[B14] Balogh Z McKinley BA Holcomb JB Miller CC Cocanour CS Kozar RA Valdivia A Ware DN Moore FA Both primary and secondary abdominal compartment syndrome can be predicted early and are harbingers of multiple organ failure J Trauma 2003 54 848 859 10.1097/01.TA.0000070166.29649.F3 12777898

[B15] McNelis J Marini CP Jurkiewicz A Fields S Caplin D Stein D Ritter G Nathan I Simms HH Predictive factors associated with the development of abdominal compartment syndrome in the surgical intensive care unit Arch Surg 2002 137 133 136 10.1001/archsurg.137.2.133 11822945

[B16] De Waele JJ De Laet I Malbrain ML Rational intraabdominal pressure monitoring: how to do it? Acta Clin Belg Suppl 2007 62 16 25 24881697 10.1179/acb.2007.62.s1.004

[B17] Cheatham ML Safcsak K Is the evolving management of intra-abdominal hypertension and abdominal compartment syndrome improving survival? Crit Care Med 2010 38 402 407 10.1097/CCM.0b013e3181b9e9b1 20095067

[B18] Malbrain ML Cheatham ML Kirkpatrick A Sugrue M Parr M De Waele JJ Balogh Z Leppaniemi A Olvera C Ivatury R D'Amours S Wendon J Hillman K Johansson K Kolkman K Wilmer A Results from the international conference of experts on intra-abdominal hypertension and abdominal compartment syndrome. I. Definitions Intensive Care Med 2006 32 1722 1732 10.1007/s00134-006-0349-5 16967294

[B19] Ejike JC Mathur M Moores DC Abdominal compartment syndrome: focus on the children Am Surg 2011 77 72 77 21944457

[B20] Ejike JC Newcombe J Baerg J Bahjri K Mathur M Understanding of abdominal compartment syndrome among pediatric healthcare providers Crit Care Res Pract 2010 doi: 10.1155/2010/876013 10.1155/2010/876013 PMC2958672 20981270

[B21] Newcombe J Mathur M Bahjiri K Ejike JC Pediatric critical care nurses' experience with abdominal compartment syndrome Annals of Intensive Care in press 10.1186/2110-5820-2-S1-S6 PMC3390293 22873422

[B22] Kimball EJ Rollins MD Mone MC Hansen HJ Baraghoshi GK Johnston C Day ES Jackson PR Payne M Barton RG Survey of intensive care physicians on the recognition and management of intra-abdominal hypertension and abdominal compartment syndrome Crit Care Med 2006 34 2340 2348 10.1097/01.CCM.0000233874.88032.1C 16878034

[B23] Akhobadze G Chkhaidze M Kanjaradze D Tsirkvadze I Ukleba V Identification, management and complications of intra-abdominal hypertension and abdominal compartment syndrome in neonatal intensive care unit (a single centre retrospective analysis) Georgian Med News 2011 58 64 21525540

[B24] Beck R Halberthal M Zonis Z Shoshani G Hayari L Bar-Joseph G Abdominal compartment syndrome in children Pediatr Crit Care Med 2001 2 51 56 10.1097/00130478-200101000-00011 12797889

[B25] Ejike JC Humbert S Bahjri K Mathur M Outcomes of children with abdominal compartment syndrome Acta Clin Belg Suppl 2007 141 148 17469712

[B26] Pearson EG Rollins MD Vogler SA Mills MK Lehman EL Jacques E Barnhart DC Scaife ER Meyers RL Decompressive laparotomy for abdominal compartment syndrome in children: before it is too late J Pediatr Surg 2010 45 1324 1329 10.1016/j.jpedsurg.2010.02.107 20620339

[B27] Kirkpatrick AW Brenneman FD McLean RF Rapanos T Boulanger BR Is clinical examination an accurate indicator of raised intra-abdominal pressure in critically injured patients? Can J Surg 2000 43 207 211 10851415 PMC3695163

[B28] Sugrue M Bauman A Jones F Bishop G Flabouris A Parr M Stewart A Hillman K Deane SA Clinical examination is an inaccurate predictor of intraabdominal pressure World J Surg 2002 26 1428 1431 10.1007/s00268-002-6411-8 12297912

[B29] Kimball EJ Kim W Cheatham ML Malbrain ML Clinical awareness of intra-abdominal hypertension and abdominal compartment syndrome in 2007 Acta Clin Belg Suppl 2007 1 66 73 24881702 10.1179/acb.2007.62.s1.009

[B30] Malbrain ML De Laet I Van RN Schoonheydt K Dits H Can the abdominal perimeter be used as an accurate estimation of intra-abdominal pressure? Crit Care Med 2009 37 316 319 10.1097/CCM.0b013e318192678e 19050639

[B31] Steinau G Kaussen T Bolten B Schachtrupp A Neumann UP Conze J Boehm G Abdominal compartment syndrome in childhood: diagnostics, therapy and survival rate Pediatr Surg Int 2011 27 399 405 10.1007/s00383-010-2808-x 21132501

[B32] De Keulenaer BL Regli A Malbrain ML Intra-abdominal measurement techniques: is there anything new? Am Surg 2011 77 Suppl 1 S17 S22 21944447

[B33] Malbrain ML Different techniques to measure intra-abdominal pressure (IAP): time for a critical re-appraisal Intensive Care Med 2004 30 357 371 10.1007/s00134-003-2107-2 14730376

[B34] Mayberry JC Goldman RK Mullins RJ Brand DM Crass RA Trunkey DD Surveyed opinion of American trauma surgeons on the prevention of the abdominal compartment syndrome J Trauma 1999 47 509 513 10.1097/00005373-199909000-00012 10498305

[B35] De Laet I Hoste EA De Waele JJ Survey on the perception and management of the abdominal compartment syndrome among Belgian surgeons Acta Chir Belg 2007 107 648 652 18274178 10.1080/00015458.2007.11680140

[B36] Tiwari A Myint F Hamilton G Recognition and management of abdominal compartment syndrome in the United Kingdom Intensive Care Med 2006 32 906 909 10.1007/s00134-006-0106-9 16601965

[B37] Kirkpatrick AW Laupland KB Karmali S Bergeron E Stewart TC Findlay C Parry N Khetarpal S Evans D Spill your guts! Perceptions of Trauma Association of Canada member surgeons regarding the open abdomen and the abdominal compartment syndrome J Trauma 2006 60 279 286 10.1097/01.ta.0000205638.26798.dc 16508483

[B38] Ravishankar N Hunter J Measurement of intra-abdominal pressure in intensive care units in the United Kingdom: a national postal questionnaire study Br J Anaesth 2005 94 763 766 10.1093/bja/aei117 15764629

[B39] Zhou JC Zhao HC Pan KH Xu QP Current recognition and management of intra-abdominal hypertension and abdominal compartment syndrome among tertiary Chinese intensive care physicians J Zhejiang Univ Sci B 2011 12 156 162 10.1631/jzus.B1000185 21265048 PMC3030961

[B40] Nagappan R Ernest D Whitfield A Recognition and management of intra-abdominal hypertension and abdominal compartment syndrome Crit Care Resusc 2005 7 298 302 16539585

[B41] Corcos AC Sherman HF Percutaneous treatment of secondary abdominal compartment syndrome J Trauma 2001 51 1062 1064 10.1097/00005373-200112000-00006 11740251

[B42] Davis PJ Koottayi S Taylor A Butt WW Comparison of indirect methods of measuring intra-abdominal pressure in children Intensive Care Med 2005 31 471 475 10.1007/s00134-004-2539-3 15678316

[B43] Suominen PK Pakarinen MP Rautiainen P Mattila I Sairanen H Comparison of direct and intravesical measurement of intraabdominal pressure in children J Pediatr Surg 2006 41 1381 1385 10.1016/j.jpedsurg.2006.04.030 16863841

[B44] Yaster M Scherer TL Stone MM Maxwell LG Schleien CL Wetzel RC Buck JR Nichols DG Colombani PM Dudgeon DL Prediction of successful primary closure of congenital abdominal wall defects using intraoperative measurements J Pediatr Surg 1989 24 1217 1220 10.1016/S0022-3468(89)80554-8 2531789

[B45] Latenser BA Kowal-Vern A Kimball D Chakrin A Dujovny N A pilot study comparing percutaneous decompression with decompressive laparotomy for acute abdominal compartment syndrome in thermal injury J Burn Care Rehabil 2002 23 190 195 10.1097/00004630-200205000-00008 12032369

[B46] Sharpe RP Pryor JP Gandhi RR Stafford PW Nance ML Abdominal compartment syndrome in the pediatric blunt trauma patient treated with paracentesis: report of two cases J Trauma 2002 53 380 382 10.1097/00005373-200208000-00034 12169953

[B47] Okhuysen-Cawley R Prodhan P Imamura M Dedman AH Anand KJ Management of abdominal compartment syndrome during extracorporeal life support Pediatr Crit Care Med 2007 8 177 179 10.1097/01.PCC.0000257102.53488.5E 17273121

[B48] Cheatham ML Safcsak K Percutaneous catheter decompression in the treatment of elevated intra-abdominal pressure Chest 2011 140 1428 1435 10.1378/chest.10-2789 21903735

[B49] De Waele JJ Hoste EA Malbrain ML Decompressive laparotomy for abdominal compartment syndrome - a critical analysis Crit Care 2006 10 R51 10.1186/cc4870 16569255 PMC1550894

[B50] Cheatham ML Malbrain ML Kirkpatrick A Sugrue M Parr M De Waele JJ Balogh Z Leppaniemi A Olvera C Ivatury R D'Amours S Wendon J Hillman K Wilmer A Results from the international conference of experts on intra-abdominal hypertension and abdominal compartment syndrome. II. Recommendations Intensive Care Med 2007 33 951 962 10.1007/s00134-007-0592-4 17377769

