# Supplementary material for: Recognition and management of abdominal compartment syndrome among German pediatric intensivists: results of a national survey
Source: Ann Intensive Care. 2012 Jul 5;2(Suppl 1):S8. doi: 10.1186/2110-5820-2-S1-S8 (PMC3390295; doi:10.1186/2110-5820-2-S1-S8)
Supplement: Additional file 1 — Postal questionnaire. [file 2110-5820-2-S1-S8-S1.docx]

# Postal questionnaire

*Part A: description of answering ICU*

A.1 Your ICU might be described as □ pure NICU

□ pure PICU

□ NICU rather than PICU □ PICU rather than NICU

If (at least *inter alia*) neonatologic ICU, what is the level of provided medical care?

□ Highest level

□ Intermediate level

□ Low level

A.2 How many patients were treated at your ICU in 2009?

□ up to 350

□ 351 to 700

□ more than 700

- of these: neonatologic share: ____________ [free text]

- of these: pediatric share: _____________ [free text]

A.3 Your ICU is conducted by □ pediatricians

□ pediatric surgeons

□ others: _________________ [free text]

A.4 □ University hospital □ non-university hospital

*Part B: intra-abdominal hypertension and abdominal compartment syndrome*

B.1 Does IAH/ACS play a role in your clinical practice? □ No

□ Yes, seldom

□ Yes, regularly

□ Yes, often

In which year was IAH/ACS diagnosed for the very first time at your ICU? _____ [free text]

B.2 How would you define IAH in children? ______________________ [free text]

How would you define ACS in children? ______________________ [free text]

B.3 How often did you diagnose ‘IAH’ at your ICU in 2009? □ 0× □ ≤10× □ >10×

How often did you diagnose ‘ACS’ at your ICU in 2009? □ 0× □ ≤5× □ >5×

- of these: ‘primary’ origin: ____________ [free text]

- of these: ‘secondary’ origin: ____________ [free text]

- of these: origin not distinguishable: ____________ [free text]

B.4 Please estimate (in percent), how often children of the following age classes are hit by IAH and ACS at your ICU:

|  | Preterms/newborns | Sucklers/infants | Toddlers | Pupils | Adolescents |
| --- | --- | --- | --- | --- | --- |
| IAH |  |  |  |  |  |
| ACS |  |  |  |  |  |

B.5 Describe your diagnosis-making process concerning IAH and ACS (mark with cross):

|  | IAH | ACS |
| --- | --- | --- |
| *Exclusively* based on clinical symptoms |  |  |
| Based on clinical symptoms *plus* IAP measurement results |  |  |
| *Exclusively* based on IAP measurement results |  |  |

Please state three clinical symptoms (in descending order of significance) which you deem most important when diagnosing IAH/ACS [free text]:

(1) ________________ (2) ________________ (3) ________________

B.6 Do you measure intra-abdominal pressures (IAP) regularly?

-No, because □ we exclusively diagnose IAH and ACS clinically

□ no adequate technical equipment is available

□ of its technical expenditure

□ of its personnel expenditure

□ IAH would not have any therapeutical consequence if

proved

□ of its accompanying danger of infection

□ of its invasiveness

-Yes, □ but only in cases of clinical signs of IAH and ACS □ but only in cases of organ dysfunction and/or failure

□ once per day

□ two times per day

□ three to four times per day

□ continuously or more than times per day, respectively

B.7 If yes, which method do you use to measure intra-abdominal pressures?

□ Directly via intra-abdominal placed pressure probes

Which type? _________________ [free text]

□ Indirectly via □ intra-vesical pressure measurement (bladder

pressure)

□ intra-gastric pressure measurement

□ intra-rectal pressure measurement

□ peak inspiratory pressure measurement

□ others: __________________ [free text]

B.8 Would you measure intra-abdominal pressure more often if handling would become easier and more standardized (e.g., comparable to CVP measurements)?

□ Yes □ No

B.9 Please name three disease patterns which to your experience most often induce IAH and ACS dependent on the age group (free text):

|  | Preterms/newborns | Sucklers/infants | Toddlers | Pupils | Adolescents |
| --- | --- | --- | --- | --- | --- |
| 1st cause |  |  |  |  |  |
| 2^nd^ cause |  |  |  |  |  |
| 3^rd^ cause |  |  |  |  |  |

B.10 At which IAP value would you induce medical (non-invasive), interventional-decompressive or surgical-decompressive therapy options dependent on the age group [free text]?

|  | Preterms/newborns | Sucklers/infants | Toddlers | Pupils | Adolescents |
| --- | --- | --- | --- | --- | --- |
| IAP > 5 mmHg |  |  |  |  |  |
| IAP > 10 mmHg |  |  |  |  |  |
| IAP > 15 mmHg |  |  |  |  |  |

B.11 Which condition would make you decide to surgically decompress children with IAH?

□ Persisting IAP > 10mmHg □ without signs of organ dysfunction(s)

□ *plus* signs of organ dysfunction(s)

□ Persisting IAP > 15mmHg □ without signs of organ dysfunction(s)

□ *plus* signs of organ dysfunction(s)

□ Persisting IAP > 20mmHg □ without signs of organ dysfunction(s)

□ *plus* signs of organ dysfunction(s)

B.12 How often did you perform surgical decompressions among children suffering from ACS at your clinic in 2009? ________ [free text]

Please estimate the survival rate of children suffering from ACS at your clinic in 2009:

(A) After decompressive laparotomy: ________ [free text]

(B) Without decompressive laparotomy: ________ [free text]

Would you again perform decompressive laparotomy in cases of ACS? □ Yes □ No
